# Commentary: Identification of diverse astrocyte populations and their malignant analogs

**DOI:** 10.3389/fnmol.2017.00193

**Published:** 2017-06-13

**Authors:** Chang-Hoon Cho

**Affiliations:** College of Public Health, Korea UniversitySeoul, South Korea

**Keywords:** astrocyte, diversity, Aldh1l1, CD51, CD71, CD63

Astrocytes, the most abundant type of cells in the nervous system, represent a population of complex and functionally diverse cells (Prochiantz and Mallat, 1988; Wilkin et al., 1990). It has been known since Ramon y Cajal's observation that they are heterogeneous across different brain regions as well as within the same brain regions (García-Marín et al., 2007). Astrocytes differ in their morphology (e.g., protoplasmic and fibrous), developmental origin (subventricular and hippocampal subgranular zones), gene expression profile (e.g., ion channels and transporters), astroglial coupling (e.g., gap junction proteins), and electrophysiological properties (e.g., membrane potential and potassium buffering) (Zhang and Barres, 2010; Ben Haim and Rowitch, 2017). The diverse functions of astrocytes include maintaining the blood-brain barrier, regulating regional blood flow, providing trophic, and metabolic factors to neurons, recycling neurotransmitters (uptake and release), maintaining homeostasis of neural networks, and regulating synaptogenesis and synaptic transmission (Khakh and Sofroniew, 2015). In the case of injuries (e.g., stroke and traumatic brain injury) and/or neurological and psychiatric diseases (e.g., Alzheimer's, Huntington's, Parkinson's, multiple sclerosis, and amyotrophic lateral sclerosis), astrocytes undergo diverse changes (e.g., reactive gliosis) that may be protective or causative with regard to pathologic phenotypes in a context dependent manner. Therefore, elucidating the mechanisms that expand the functional diversity of astrocytes and identifying the intrinsic and extrinsic factors for astrocytic heterogeneity have been a hot topic in glia research.

A recent report published by Drs. Benjamin Deneen and Chad Creighton at the Baylor College of Medicine has identified five distinct astrocyte subpopulations by collecting astrocytes from adult brains of ALDH1L1-eGFP mice (3–4 months old) using FACS-based techniques (John Lin et al., 2017). They selected three (CD51, CD71, and CD63) out of 81 cell surface antigens screened as markers to categorize astrocytes into distinct populations (A–E). Then, they isolated astrocytes from five different brain areas and compare the distribution of those populations. CD51, integrin alpha V protein (encoded by ITGAV gene), has been shown to be involved in maturation and migration of astrocytes and neuron–astrocyte interactions (Milner et al., 1999; Hermosilla et al., 2008). Increased expression of CD51 has been shown in glioblastoma (Roth et al., 2013). CD63, also called LAMP-3 and tetraspanin-30, is a lysosomal marker and it is involved in gliotransmitter release from astrocytes via Ca^2+^ dependent exocytosis (Li et al., 2008). Increased expression of CD63 has been shown in astrocytoma (Rorive et al., 2010). CD71, transferrin receptor 1 (encoded by TFRC gene), has an increased expression in astrocytes under hypoxic condition and in glioblastoma (Yang et al., 2012; Verbovšek et al., 2014). They found that population C (CD51^+^CD71^−^CD63^−^) astrocytes, the most abundant in all brain regions examined, expressed synapse-related genes to promote the neuronal synaptogenesis and synapse function. They examined this by measuring the increased expression of presynaptic and postsynaptic markers, and increased frequency of excitatory and inhibitory postsynaptic currents in neurons when co-cultured with population C astrocytes. Population A (CD51^−^CD71^−^CD63^−^) astrocytes were enriched region-specifically in the thalamus and olfactory bulbs. They also found that population C is more proliferative than population A. In contrast, population A displayed more migratory potentials than population C. The distribution of five astrocyte populations from two mouse models of malignant glioma that the authors developed were examined. Populations C and D (CD51^−^CD71^+^CD63^−^) were proportionally larger in population than other populations, most likely because those glioma models show severe symptoms (increased neuronal excitability and seizures) due to the increased aberrant growth of synapses. Human glioblastoma/glioma specimens were also examined for the distribution of these populations and found that population A was the majority in the limited cases shown in this study.

Many questions are raised from this interesting study. First, does this astrocytic classification cover all the astrocyte populations in the nervous system? Although Aldh1l1 has been known to be one of best astrocyte markers, other markers such as GFAP, GLAST, GLT1, GS, and AQP4 are also recognized as astrocyte-specific (Yang et al., 2011). Therefore, it may need to be examined whether isolated astrocytes from other transgenic mice expressing fluorescent proteins under other astrocyte-specific promoters could follow this classification of astrocytes. This study showed that the distribution of astrocytic subpopulations is not static. Population E (CD51^−^CD71^−^CD63^+^) is the majority in the cortex at P28. In contrast, this population is the minority (10%) at the age of 3–4 months old. CD71's expression has been shown to be drastically decreased in aged mice (13 months; Duan et al., 2009). This means that CD71 may lose the capacity as a good marker for astrocyte classification beyond the young adult stages. Therefore, this classification needs to be examined as to whether it holds over the entire lifespan of animals. Otherwise, it cannot be used to classify astrocytes in old animals which may be critical for age-related research (e.g., Alzheimer's disease). It is also possible that further classification within the same subpopulation or novel class(es) of astrocytes (with alternative surface markers) can emerge over time throughout the animals' lifespan. This might be addressed by using these three markers to sort astrocytes from the animals (e.g., CD51-eGFP mice) and examining the gene expression profiles.

Second, can this astrocyte classification be useful in injury and disease models? In these models where astrocytes undergo phenotypic changes, would these astrocyte populations play distinctive roles? Would these astrocyte populations respond differently when challenged with TGFß, FGF, or LPS either in primary culture systems or animal models *in vivo?* In these models and conditions, would secreted factors (e.g., GPC4/6, SPARCL1 or THBS1/2) from astrocytes differ from each population so that they have different effects on various neurons (and other astrocytes)? Would astrocyte subpopulations respond differentially to microglial secreted factors (e.g., IL-1α, TNF, and C1q) in inflammation or in brain disease models (Liddelow et al., 2017)?

Lastly, can this classification be traced back to the progenitors following the lineage over the embryonic period (Schitine et al., 2015)? Are distinct astrocyte populations determined at early developmental stages or they are established locally after reaching the particular brain regions (or both)?

## Author contributions

The author confirms being the sole contributor of this work and approved it for publication.

### Conflict of interest statement

The author declares that the research was conducted in the absence of any commercial or financial relationships that could be construed as a potential conflict of interest.
